# Effects of mixed legume leaf powder supplementation on nutrient intake, blood metabolites, and reproductive hormone profiles in buffaloes

**DOI:** 10.14202/vetworld.2026.1611-1628

**Published:** 2026-04-25

**Authors:** Yeni Widiawati, Lisa Praharani, Diana Andrianita Kusumaningrum, Tatan Kostaman, Santiananda Arta Asmarasari, Eko Handiwirawan, Dwi Yulistiani, Endang Romjali, Chalid Talib, Maureen Chrisye Hadiatry, Priyono Priyono, Moyosore Joseph Adegbeye

**Affiliations:** 1 Research Center for Animal Husbandry, Research Organization for Agricultural and Food, National Research and Innovation Agency, Cibinong Science Center, Bogor, Indonesia; 2 Research Center for Behavioral and Circular Economics, National Research and Innovation Agency, Jakarta, Indonesia; 3 Department of Animal Production and Health, University of Africa, Toru-Orua, Bayelsa-State, Nigeria

**Keywords:** amino acid, buffalo, digestibility, growth performance, mixed legumes, nutrient intake, reproductive hormones, supplementation

## Abstract

**Background and Aim:**

Buffaloes play a vital role in tropical livestock systems, yet their productivity and reproductive efficiency are strongly influenced by nutritional management. Protein- and amino acid-rich legume forages have been widely proposed as sustainable dietary supplements to enhance metabolic and reproductive performance. However, evidence regarding their effectiveness in animals maintained under adequate nutritional status remains limited. Therefore, this study evaluated the effects of supplementation with mixed legume leaf powder on nutrient intake, digestibility, blood metabolites, growth performance, and reproductive hormone profiles in female buffaloes.

**Materials and Methods:**

Sixteen female swamp buffaloes (initial body weight 532.85 ± 85.58 kg) were assigned to control and treatment groups in a completely randomized design. The control group received a diet of Napier grass and concentrate, whereas the treatment group received an additional 10% mixed legume leaf powder comprising *Gliricidia sepium, Calliandra calothyrsus*, and *Leucaena leucocephala* (1:1:1). Feed intake, apparent digestibility, and amino acid intake were measured. Blood samples were collected to determine glucose and urea concentrations. Reproductive hormone profiles (progesterone and estrogen) were monitored throughout the estrous cycle. Data were analyzed using analysis of variance and linear mixed-effects models.

**Results:**

Supplementation with mixed legume leaf powder significantly increased intake of dry matter, organic matter, crude protein, gross energy, and most amino acids (p < 0.05). Digestibility of crude protein, as well as digested intake of dry matter and organic matter, was also significantly improved (p < 0.05). However, serum urea and glucose concentrations were not affected by dietary treatment (p > 0.05). Similarly, progesterone and estrogen profiles across the estrous cycle showed no significant differences between control and treatment groups (p > 0.05). Body weight gain did not improve significantly despite increased nutrient intake.

**Conclusion:**

Mixed legume leaf powder supplementation enhances nutrient intake, amino acid supply, and digestibility in female buffaloes but does not influence metabolic status, growth performance, or reproductive hormone dynamics under adequate nutritional conditions. These findings suggest that additional protein supplementation may provide limited benefits when animals are already maintained under optimal feeding and body condition. Further studies under nutrient-deficient conditions and over longer durations are recommended to clarify potential reproductive advantages.

## INTRODUCTION

Buffaloes constitute an integral component of Indonesian sociocultural and agricultural systems, contributing to meat and milk production, draft power, and traditional practices. They are well adapted to hot and humid environments and possess several advantages over other ruminants, including efficient utilization of high-fiber diets, resilience to extreme climatic conditions, and enhanced disease resistance [1–3]. Despite these favorable attributes, the buffalo population in Indonesia has declined markedly from 1.133 million in 2019 to 556,794 in 2024, representing a 50.89% reduction over 5 years [4]. One of the primary constraints on population growth is the reproductive characteristics of female buffaloes, including delayed puberty, increased age at first calving, silent estrus, and prolonged postpartum anestrus [5–7].

Nutritional management plays a pivotal role in improving reproductive performance in buffaloes, particularly under tropical smallholder conditions where feed resources are frequently limited in both quality and quantity. Inadequate nutrition has been linked to reduced growth, extended calving intervals, and reproductive disorders such as anestrus [8]. Supplementation with balanced diets containing adequate protein, minerals, and vitamins has been demonstrated to enhance fertility, regulate hormonal balance, and support postpartum recovery in female buffaloes and dairy cattle [9, 10]. Recent studies have emphasized that protein-rich legumes, including *Leucaena leucocephala*, *Gliricidia sepium*, and *Calliandra calothyrsus*, serve as valuable alternative feed resources for tropical buffaloes by improving nitrogen balance, growth performance, and metabolic profiles associated with reproductive efficiency [11–13]. The elevated crude protein content and essential amino acids (e.g., arginine and leucine) present in these forages contribute to follicular development, conceptus growth, and hormonal regulation. Concurrently, mineral and vitamin supplementation further enhances reproductive performance [14, 15]. Although substantial evidence supports the role of legume supplementation and specific amino acids in improving productivity and metabolic health in ruminants, direct evidence linking these nutrients to reproductive outcomes in buffaloes, such as estrus expression, conception rate, and calving interval, remains limited, thereby highlighting a significant research gap requiring controlled investigation under tropical smallholder conditions.

Despite growing evidence supporting the role of protein-rich legumes in improving nutrient intake and metabolic status in ruminants, several important gaps remain in the current literature. First, most studies evaluating legume supplementation have primarily focused on growth performance, nitrogen utilization, and general metabolic responses, whereas detailed investigations linking dietary protein and amino acid intake to reproductive endocrine regulation in buffaloes remain limited [11–13]. Second, existing research has largely been conducted under conditions of nutritional deficiency or feed restriction, where animals exhibit more pronounced metabolic responses, thereby limiting the applicability of these findings to well-managed production systems [1, 8]. Third, studies that integrate nutrient intake, amino acid composition, blood metabolites, and reproductive hormone dynamics within a single experimental framework remain scarce, particularly in swamp buffaloes. Moreover, the assumption that increased dietary protein consistently enhances reproductive performance has been questioned because endocrine responses may plateau when animals are maintained under adequate nutritional conditions and optimal body condition [16]. In addition, mixed legume leaf supplementation combining *L. leucocephala*, *G. sepium*, and *C. calothyrsus* has not been sufficiently evaluated for its combined effects on metabolic and reproductive physiology. These limitations highlight the need for controlled studies that simultaneously assess nutrient utilization, metabolic indicators, and reproductive hormone profiles under conditions reflecting practical feeding systems.

Therefore, this study was designed to evaluate the effects of supplementation with mixed legume leaf powder on nutrient intake, apparent digestibility, blood metabolites, growth performance, and reproductive hormone profiles in female swamp buffaloes maintained under adequate nutritional management. Specifically, the study aimed to determine whether increased dietary protein and amino acid intake derived from a mixture of *L. leucocephala*, *G. sepium*, and *C. calothyrsus* could influence metabolic status, as reflected by serum urea and glucose concentrations, and reproductive endocrine responses, as indicated by progesterone and estrogen profiles throughout the estrous cycle. In addition, this study sought to clarify whether dietary protein enrichment provides additional physiological benefits in animals already maintained under good body condition, thereby contributing to evidence-based feeding strategies for buffalo production systems.

## MATERIALS AND METHODS

### Ethical approval

The experiment was conducted in strict accordance with the ethical guidelines for the care and use of animals in research. All procedures involving the use of live animals were reviewed and approved by the Animal Ethics Committee of the Agency for Agricultural Research and Development, Ministry of Agriculture, Republic of Indonesia. The study received formal ethical approval under registration number Balitbangtan/Balitnak/Rm/ 01/2021.

The approval covered all aspects of the experimental protocol, including animal selection, housing conditions, feeding management, health monitoring, blood sampling, estrus synchronization using prosta-glandin, and any other interventions performed during the 120-day study period. The committee ensured that the procedures complied with national regulations and international standards for animal welfare, with particular emphasis on the principles of the 3Rs (Replacement, Reduction, and Refinement).

This ethical approval confirms that the study was designed and executed with full consideration for animal welfare, and that the scientific objectives were balanced with the highest standards of humane treatment.

### Study period and location

The study was conducted from March to December 2021 at the Indonesian Research Center for Animal Production (IRIAP) in Bogor, Indonesia. The IRIAP is located in Banjarwaru Village, Ciawi Subdistrict, Bogor Regency, West Java, Indonesia, at approximately 6.63°–6.65° S latitude and 106.78°–106.80° E longitude, with an elevation of about 490–510 m above sea level. The area has a humid tropical climate. Mean daily air temperatures generally range from 26°C to 31°C. Relative humidity remains high throughout the year, typically ranging from 80% to 90%.

### Animals and housing management

The experiment used eighteen female swamp buffaloes (*Bubalus bubalis*). The number of animals was limited by availability at the research station. The buffaloes had similar ages, parities, and body condition scores (BCS). Their average initial body weight was 532.85 ± 85.58 kg. All animals were in the post-second-calving stage and had remained unmated for 1 year after parturition. Their ages ranged from 6 to 8 years, and none had reproductive abnormalities or diseases. The BCS of the buffaloes was assessed on a five-point scale (1.0 = extremely thin to 5.0 = extremely fat), with values ranging from 3.0 to 3.5, indicating moderate to good body condition.

The animals were housed individually in pens measuring approximately 2 m × 2.5 m in an open-sided shed with an asbestos roof and a concrete floor. Each pen had separate feed and watering channels. Drinking water was available *ad libitum* and was cleaned every morning. Daily management was standardized throughout the experiment. The experimental animals were housed individually without forced exercise and were handled by the same trained personnel during feeding, sampling, and pen cleaning to minimize stress. Pens were cleaned daily using procedures that avoided disturbing the animals or inducing stress.

All animals were clinically monitored before, during, and after the experiment. Before the experiment, all animals were treated with an anthelmintic (Kalbazen, 10 mg/kg body weight, orally) and vitamins (injectable vitamins A, D_3_, and E (Vigantol E, PT. SHS International, Jakarta, Indonesia) at 1 mL per 50 kg body weight, intramuscularly). Animal health was monitored weekly throughout the experimental period. All health treatments followed the procedures approved by the Animal Ethics Commission. Two buffaloes were withdrawn during the last observation period due to sickness (one from the control group and one from the treatment group), and their data were excluded from the final analysis. Therefore, statistical analyses were performed using data from 16 buffaloes, which constituted the experimental units included in the study. All measured variables were analyzed using these 16 animals to ensure consistency and reliability in the statistical evaluation.

### Experimental design

The animals were grouped by initial body weight and age, then randomly assigned to dietary treatments using a random-number method to ensure balanced groups and a normal distribution. Two groups of animals received dietary treatments in a completely randomized design, as the experimental animals were relatively homogeneous in age, parity, body weight, and BCS. The control group consisted of nine buffaloes with a mean body weight of 536.17 ± 88.57 kg and was fed fresh chopped Napier grass and concentrate in a ratio of 60.9%:39.1% on a dry matter (DM) basis. The treatment group consisted of nine buffaloes (512 ± 77.37 kg) and was fed fresh, chopped Napier grass, concentrate, and a supplement of a mixture of three legume leaf powders (*G. sepium*, *C. calothyrsus*, and *L. leucocephala*) in a 1:1:1 ratio (MLP). The diet composition was 54.5% Napier grass, 35.1% concentrate, 10.4% MLP on a DM basis.

The experimental design followed a 120-day timeline, which included an initial adaptation period (acclimatization to the experimental feed) of 26 days, body weight gain measurement for 56 days, feed intake and digestibility measurements for 7 days, metabolite assessment for 1 day, and hormone profiling for 30 days.

### Feed and feeding management

The MLP was prepared by drying *Leucaena*, *Calliandra*, and *Gliricidia* under the sun at an average ambient temperature of 29°C–31°C for 3 days, or until dry (DM 85%), then grinding the material using a hammer mill with a 5 mm sieve. The purpose of drying was to reduce anti-nutritional factors such as mimosine, coumarin, and tannins [17]. The resulting powder was stored under refrigerated conditions (4°C) for approximately one month prior to use in the experiment. Drying was conducted stepwise, and samples were collected at each stage to determine moisture content and nutrient composition. Each prepared legume was mixed in a 1:1:1 ratio to form the MLP used for the feeding treatment.

Initially, the experimental diets were formulated to be iso-nitrogenous (11% crude protein [CP]) and iso-energetic (19 MJ/kg of GE). However, because the feeds were not provided as a total mixed ration and the leguminous forages were sourced from areas surrounding smallholder farms with varying stages of maturity, the average nutritional quality of the diets during the experimental period slightly deviated from the original formulation. As a result, the treatment diets contained approximately 13% CP and 21.6 MJ/kg GE. Consequently, the present study primarily focused on evaluating the effects of feeding a combination of three shrub legume species on nutrient intake, digestibility, and reproductive hormones in female buffaloes.

The animals were adapted to their experimental diets over 30 days. On a DM basis, the feeds offered to the control group were 4.9 kg of chopped Napier grass plus 4.4 kg of concentrate, while animals in the treatment group were offered 4.9 kg of chopped Napier grass plus 4.4 kg of concentrate plus 0.92 kg of MLP. The formulated concentrate provided approximately 18.7% CP and consisted of locally available feed ingredients; its composition is presented in Table 1.

**Table 1:** Concentrate proportion (%) and the nutrient content, on a dry matter basis of feeds used in the experiment.

Ingredients	% proportion	Napier grass	legume leaves mixture
Ground corn	33.9		
Rice bran	20		
Soy bean meal	24.1		
Copra meal	10		
Palm kernel meal	5		
Molasses	5		
Mineral premix	2		
Nutrient content (%)			
Crude protein	18.72	6.43	26.04
Crude fat	7.39	1.58	2.72
Crude fiber	15.28	39.78	20.78
Ash	9.30	13.66	5.12
Calcium	1.41	0.24	1.04
Phosphorus	0.44	0.32	0.20
Gross energy (Kcal/kg)	4042	3592	4430

The mineral premix (MixPro-Kalbe Indonesia) per kg primarily contains vitamin A 1,000,000 IU; vitamin D 188,000 IU; phosphorus 13,200 mg; calcium 22,000 mg; magnesium sulfate 10,000 mg; and other minerals, vitamins, and amino acids.

The animals were fed fresh chopped Napier grass (*Pennisetum purpureum*) as a basal diet. The daily feed was divided into two equal portions and provided twice a day at 8:00 a.m. and 2:00 p.m. The feed was offered to the animals at approximately 3% of body weight on a DM basis to achieve *ad libitum* intake. Daily adjustments were made to maintain refusals of approximately 8%–10% of the total feed offered [18, 19]. The amount of feed offered and refused the next day was recorded daily to determine feed intake. Samples of feed offered and refused were collected daily, weighed, oven-dried, and ground to pass through a 3 mm screen for further analysis of DM, organic matter (OM), CP, and GE The contents of OM, CP, crude fat, GE, CF, ash, Ca, and P of the feeds used in the experiment are shown in Table 1. Table 2 presents the ingredients and chemical compositions of the diets used during the experiment. Diets were formulated based on the nutrients required for maintaining female buffaloes, following the recommendations of Faraz and Waheed [20]. The nutrient requirements reported by Faraz and Waheed [20] were used as a reference to ensure that the experimental diets met the maintenance requirements of buffalo heifers. It should be noted that the diets described by Faraz and Waheed [20] were not formulated to be iso-nitrogenous or iso-energetic. In contrast, the experimental diets in the present study were initially designed to be iso-nitrogenous and iso-energetic, with additional amino acid sources from legume leaves, to evaluate their effects on reproductive hormone profiles.

**Table 2:** The ingredients and chemical composition of two different diets.

Items	Dietary treatment	Control
Ingredients (% of DM)		
Napier grass	54.5	60.9
Concentrate	35.1	39.1
Legume leaves powder (MLP)	10.4	0
Chemical composition (% of DM)		
Organic matter	88.75	88.05
Crude protein	13.05	11.10
Gross energy (Kcal/kg)	51680	45500
Crude fiber	29.22	30.19
Fat	3.74	3.85
Calcium	0.733	0.698
Phosphorus	0.35	0.367

### Apparent digestibility

Apparent digestibility was measured over seven days. Daily feed intake and refusals were recorded prior to the morning feeding during the digestibility trial. Fecal output was collected daily, and a representative subsample equivalent to 10% of the total fecal production was oven-dried at 60°C for 48 h. Subsamples of feed offered, refused, and feces were collected, weighed, and recorded every day. At the end of the collection period, fecal samples were pooled per animal, and a 10% subsample was ground to pass through a 1-mm sieve and stored at −20°C for subsequent analyses. Urine was not collected in the study due to limitations of the available facilities. All pooled subsamples were then used to analyze DM, OM, CP, and GE contents.

Apparent digestibility of DM, OM, and CP was calculated based on the difference between nutrient intake and fecal output using the total collection method, as described by Van Soest [21]:

DMD (%) = [DM intake (g) – DM feces (g)] / DM intake (g) × 100%

OMD (%) = [OM intake (g) – OM feces (g)] / OM intake (g) × 100%

CPD (%) = [CP intake (g) – CP feces (g)] / CP intake (g) × 100%

### Chemical analysis of feeds

The chemical analyses of the experimental diets were performed at a certified commercial laboratory for proximate analysis, including determination of DM, OM, CP, CF, and mineral contents according to methods described by the Association of Official Analytical Chemistry [22]. The GE content was analyzed at the commercial laboratory using a bomb calorimeter (Parr 1341 Oxygen, Thermalindo Indonesia). Amino acids contained in the MLP were analyzed using High Performance Liquid Chromatography (HPLC) (Wincom LC1001A, China). Since the analyses were conducted in a certified commercial laboratory, all analytical procedures followed established standard protocols, and the analyzer was calibrated with internal quality controls.

Sample analysis and data interpretation were performed in a blinded manner, with laboratory personnel and data analysts unaware of the experimental groups to minimize analytical and interpretive bias. All samples were analyzed in duplicate, with mean values used for statistical analysis.

### Blood metabolites

Blood samples for analysis of glucose and urea concentrations were collected from each animal at 0, 2, 4, 6, 8, 10, and 12 h post-feeding (morning feeding time), following the procedure outlined by Ortolani *et al*. [23]. Blood samples were collected from the jugular vein using heparinized vacutainers. The blood samples were then immediately taken to the laboratory for glucose and urea analyses using a Chemistry Analyzer (NB Medical System MS380 P).

### Reproductive hormone profile

Observation of the reproductive hormone profile began with estrus synchronization using two injections of prostaglandin (Lutalyse, Zoetis, USA) at 11-day intervals, followed by observation of estrus [24]. The appearance of signs of estrus was recorded as day 0 of the estrous cycle. Blood samples were collected after the second injection of prostaglandin at 3-day intervals over one month. Sampling occurred on days 1, 4, 7, 10, 13, 16, 19, and 22 of the estrous cycle. Blood samples were collected via jugular vein puncture using heparinized vacutainers (5 mL). The blood samples were centrifuged immediately (3000 × *g*, 15 min), after which the plasma was separated, placed in vials, and stored in a refrigerator (−20°C) until all samples were collected and ready for analysis. Progesterone and estrogen concentrations were determined using enzyme-linked immunosorbent assay (ELISA).

Progesterone concentrations were determined using a commercial ELISA kit (Item # 4S00121, General Biological Corps) with acceptable assay precision (intra-assay CV: 2.4–7.1%; inter-assay CV: 2.6–12.6%). The minimum detectable concentration of the Progesterone ELISA assay, as measured by 2 SD from the mean of a zero standard, was estimated to be 0.0625 ng/mL. Samples were analyzed in duplicate, diluted when necessary, and all samples from the same animal were run on the same plate to minimize inter-plate variability.

Estradiol concentrations were measured using a commercial ELISA kit (Item # 4S00071, General Biological Corps), with intra- and inter-assay CVs of 2.6–13.4% and 3.3–18.0%, respectively, indicating acceptable assay precision. The minimum detectable concentration of the Estradiol ELISA assay, as measured by 2 SD from the mean of a zero standard, was estimated to be 10 pg/mL.

### Statistical analysis

All statistical analyses were performed using Stata/MP version 16.0 (StataCorp LLC, College Station, TX, USA) to evaluate the effects of dietary treatments over time on feed intake, nutrient digestibility, blood metabolites, and reproductive hormone profiles. The effects of dietary treatments on feed intake and digestibility were analyzed by analysis of variance (ANOVA) using the general linear model procedure. Prior to performing ANOVA, the assumptions of normality and homogeneity of variances were evaluated. Normality of residuals was assessed using the Shapiro–Wilk test, while homoscedasticity was examined using Levene’s test. The statistical model included dietary treatment as a fixed effect. The model did not include body weight, parity, or BCS as variables because of the relative homogeneity across animals. The model was as follows:

Y_ij_ = μ + T_i_ + e_ij_

Where Y_ij_ is the dependent variable (e.g., feed intake and digestibility), μ is the overall treatment mean, T_i_ is the fixed effect of the diet, and e_ij_ is the random error. Tukey’s test was used to compare the treatment means for the two treatments.

For reproductive hormone concentrations measured repeatedly over time in the same animals, data were analyzed using linear mixed-effects models to account for within-animal correlation. Dietary treatment, sampling time, and their interaction (diet × time) were included as fixed effects, while animal was included as a random effect. Time was treated as a repeated measure. The statistical model was specified as:

Y_ij_ = μ + T_i_ + Time_j_ + (T × Time)_ij_ + Animal + e_ij_

Where Y_ij_ is the dependent variable (hormone concentration); μ is the overall mean, T_i_ is the fixed effect of dietary treatment, Time_j_ is the fixed effect of sampling time, (T × Time)_ij_ is the interaction between diet and time, Animal is the random effect of the animal, and e_ij_ is the residual error.

The inclusion of animal as a random effect allowed the model to account for inter-individual variability. Residual diagnostics were performed to assess model assumptions. When significant effects were detected, post hoc comparisons between treatments and sampling times were conducted using Tukey’s adjustment to control for multiple comparisons. Statistical significance was declared at p < 0.05, and tendencies were discussed at 0.05 ≤ p < 0.10.

## RESULTS

### Chemical composition of dietary treatments and amino acids of legume mixture

Table 2 showed that the amount of MLP added to the daily rations of the treatment diet was up to 10.4% on a DM basis. This addition increased the CP content of the treatment diet up to 17.6% and the GE content by 13.58% but decreased the CF content by 3.32% compared to the control diet.

Table 3 summarizes the amino acid content of MLP in the diet. Seventeen amino acids were identified, including L-Cystine, L-Methionine, L-Serine, L-Glutamic acid, L-Phenylalanine, L-Isoleucine, L-Valine, L-Alanine, L-Arginine, Glycine, L-Lysine, L-Aspartic Acid, L-Leucine, L-Proline, L-Tyrosine, L-Threonine, and L-Histidine. The proportion of the amino acids was between 1-18.3 mg/kg, and the leaf mixture was rich in L-Glutamic acid (18.281 mg/kg), followed by L-Leucine (17.704 mg/kg) and L-Tyrosine (16.424 mg/kg). In contrast, L-methionine (1.793 mg/kg) was the least abundant amino acid.

**Table 3:** The amino acid content of MLP, concentrate, and Napier grass used as feed treatment in the experiment.

Amino acids (mg/kg DM)	Legume leaves	Concentrate	Napier grass
L-Cystine	5.619	2.700	1.200
L-Methionine	1.793	2.520	1.600
L-Serine	11.682	9.360	5.000
L-Glutamic acid	18.281	33.300	17.000
L-Phenylalanine	13.651	9.000	5.000
L-Isoleucine	9.202	8.460	4.400
L-Valine	11.213	8.280	4.800
L-Alanine	9.455	7.380	5.500
L-Arginine	13.412	12.960	5.500
Glycine	13.660	7.020	4.500
L-Lysine	12.256	11.520	4.200
L-Aspartic acid	8.051	20.700	11.000
L-Leucine	17.704	13.500	8.500
L-Tyrosine	16.424	6.120	3.500
L-Proline	7.473	8.460	5.000
L-Threonine	11.538	7.200	3.800
L-Histidine	10.524	4.860	2.300

### Effects of MLP supplementation on feed intake and digestibility

Table 4 shows the effect of MLP supplementation on buffalo performance parameters. The results indicate that the MLP supplementation did not significantly (p > 0.05) improve body weight or body weight gain. However, all parameters of feed intake (kg/day) were significantly affected (p < 0.05), whereas crude fiber and phosphorus were not. In contrast, MLP supplementation was similar in feed intake per body weight (g/day). The CP intake per metabolic weight was significant (p < 0.05).

**Table 4:** Feed intake of experimental feeds by animals in control and treatment groups during the experiment period.

Variables	Treatment	Control	p-value
Body weight (kg)			
Initial	545.50 ± 83.06	520.22 ± 87.49	0.632
Final	551.50 ± 82.00	524.00 ± 87.20	0.599
Weight gain (kg/mo)	6.00 ± 3.66	3.78 ± 3.40	0.271
Feed intake (kg/day)			
Dry matter	12.73 ± 0.36^a^	11.91 ± 0.56^b^	0.009
Organic matter	11.31 ± 0.31^a^	10.49 ± 0.51^b^	0.004
Crude protein	1.70 ± 0.02^a^	1.34 ± 0.14^b^	0.000
Gross energy (MJ/day)	204.99 ± 5.36^a^	187.91 ± 9.62^b^	0.001
Fat	0.49 ± 0.01	0.46 ± 0.01	0.233
Crude fiber	3.65 ± 0.14	3.59 ± 0.19	0.565
Calcium	0.10 ± 0.00^a^	0.08 ± 0.01^b^	0.000
Phosphorus	0.04 ± 0.00	0.04 ± 0.00	0.197
Feed intake/body weight (g/day)			
Dry matter	23.63 ± 3.41	23.48 ± 4.16	0.950
Organic matter	21.00 ± 3.04	20.67 ± 3.64	0.875
Crude protein	3.15 ± 0.48	2.64 ± 0.45	0.095
Gross energy (MJ/day)	0.381 ± 0.055	0.370 ± 0.065	0.782
Feed intake/metabolic weight (g/kg BW^0.75^)			
Dry matter	113.60 ± 1.81	111.18 ± 15.69	0.781
Organic matter	100.98 ± 10.54	97.90 ± 13.75	0.690
Crude protein	15.16 ± 1.70^a^	12.52 ± 1.79^b^	0.025
Gross energy (MJ/day)	1.83 ± 0.19	1.75 ± 0.25	0.580

a,b,c,d Means within row bearing different superscripts differ significantly (p < 0.05) Feed intake/body weight (g/day) = daily intake (g DM/day) : Body weight (kg) Feed intake/metabolic weight (g/kg BW0.75) = daily intake (g DM/day) : metabolic weight of the animal (kg BW0.75).

Table 5 shows that the MLP supplementation improved CP digestibility, CP digested (kg/day), digested per body weight (gram/day/kg BW), and digested per metabolic weight (gram/day/kg BW0.75) significantly (p < 0.05). The treatments also improved DM and OM digested significantly (p < 0.05).

**Table 5:** Apparent digestibility of experimental feeds by animals in control and treatment groups during the experiment period.

Variables	Treatment	Control	p-value
Digestibility (%)			
Dry matter (DMD)	67.93 ± 5.09	61.84 ± 6.31	0.072
Organic matter (OMD)	70.13 ± 5.09	64.04 ± 6.31	0.072
Crude protein (CPD)	79.63 ± 2.99^a^	73.43 ± 5.06^b^	0.014
Digested intake (kg/day)			
Dry matter (DDMI)	8.66 ± 0.81^a^	7.37 ± 1.00^b^	0.019
Organic matter (DOMI)	7.94 ± 0.72^a^	6.72 ± 0.91^b^	0.014
Crude protein (DCPI)	1.35 ± 0.06^a^	0.99 ± 0.17^b^	0.000
Digested intake/body weight (gram/day/kg BW)			
Dry matter (DDMI_BW)	16.05 ± 2.63	14.58 ± 3.08	0.415
Organic matter (DOMI_BW)	14.73 ± 2.40	13.30 ± 2.79	0.381
Crude protein (DCPI_BW)	2.51 ± 0.40^a^	1.95 ± 0.40^b^	0.033
Digested intake/metabolic weight (gram/day/kg BW^0.75^)			
Dry matter (DDMI_MW)	77.21 ± 10.17	69.01 ± 12.70	0.258
Organic matter (DOMI_MW)	70.85 ± 9.23	62.92 ± 11.50	0.228
Crude protein (DCPI_MW)	12.08 ± 1.47^a^	9.21 ± 1.73^b^	0.008

a,b,c,d Means within row bearing different superscripts differ significantly (p < 0.05)

### Effects of MLP supplementation on serum parameters

Table 7 shows the postprandial serum urea concentration of animals in the control and MLP treatment groups. The serum urea concentration reached a peak at 4 and 6 h post-feeding for the control and treatment groups, respectively. Serum urea concentration did not differ significantly between the control and treatment groups at any sampling time post-feeding (*p* > 0.05). In both groups, serum urea levels fluctuated slightly across the 0–12 h post-feeding period. In control groups, the highest mean value was observed at 4 h post-feeding, whereas in the treatment group, its peak was reached at 6 h post-feeding. In both groups, the lowest serum urea concentration occurred at 12 h post-feeding. The lowest serum urea concentration was observed at 12 h post-feeding among the seven blood sampling points conducted between 0 and 12 h post-feeding.

**Table 6:** Amino acid intake by female buffalo fed in experimental diets.

No.	Amino acid	Treatment (Mean ± SD)	Control (Mean ± SD)	p-value
1	L-Cystine	28.48 ± 0.43	21.34 ± 0.61	<0.001
2	L-Methionine	24.95 ± 0.57	23.38 ± 0.81	0.003
3	L-Serine	93.43 ± 1.78	80.03 ± 2.54	<0.001
4	L-Glutamic acid	294.70 ± 6.07	279.03 ± 8.65	0.005
5	L-Phenylalanine	94.49 ± 1.78	78.34 ± 2.54	<0.001
6	L-Isoleucine	81.77 ± 1.57	71.47 ± 2.24	<0.001
7	L-Valine	86.38 ± 1.71	73.52 ± 2.44	<0.001
8	L-Alanine	84.35 ± 1.96	74.34 ± 2.80	<0.001
9	L-Arginine	116.07 ± 1.96	100.54 ± 2.80	<0.001
10	Glycine	81.89 ± 1.61	65.44 ± 2.29	<0.001
11	L-Lysine	99.07 ± 1.50	84.40 ± 2.14	<0.001
12	L-Aspartic acid	181.44 ± 3.92	176.57 ± 5.60	0.112
13	L-Leucine	144.51 ± 3.03	124.73 ± 4.32	<0.001
14	L-Tyrosine	56.96 ± 1.25	36.07 ± 1.78	<0.001
15	L-Proline	83.34 ± 1.78	75.81 ± 2.54	<0.001
16	L-Threonine	75.13 ± 1.36	61.23 ± 1.93	<0.001
17	L-Histidine	52.77 ± 0.82	39.42 ± 1.17	<0.001

**Table 7:** Mean values of blood urea concentration (mg/dl) of buffalo fed on experimental diets.

After feeding time (h)	Control (Mean ± SD)	Treatment (Mean ± SD)	p-value
0	65.033 ± 8.88	64.250 ± 14.24	0.892
2	64.717 ± 8.25	66.250 ± 13.81	0.791
4	67.317 ± 7.02	66.167 ± 12.56	0.842
6	64.750 ± 8.49	68.967 ± 11.45	0.466
8	64.750 ± 5.21	64.533 ± 12.24	0.970
10	64.633 ± 5.49	61.083 ± 10.73	0.540
12	62.600 ± 5.41	60.867 ± 10.20	0.765

After feeding time: 08:00 am (0), 10:00 am (2), etc. a,b,c,d Means within column bearing different superscripts differ significantly (p < 0.05).

Table 6 illustrates that supplementation with MLP significantly increased (p < 0.05) the amino acid intake of female buffalo, but not L-aspartic acid.

Table 8 shows the postprandial serum glucose pattern of the animals fed two experimental diets. The blood glucose concentration differed significantly between the control and treatment groups at 0 h post-feeding (p < 0.05). The lower value observed at this sampling time occurred in the treatment group. However, no significant differences were detected in the serum glucose concentration between groups at subsequent sampling times (2–12 h post-feeding) (p > 0.05). In both groups, serum glucose concentration showed moderate fluctuations throughout the post-feeding period, with no consistent temporal pattern.

**Table 8:** Mean values of blood glucose concentration (mg/dl) of buffalo fed on experimental diets.

After feeding time (h)	Control (Mean ± SD)	Treatment (Mean ± SD)	p-value
0	58.33 ± 21.80	38.50 ± 14.20	0.017
2	53.83 ± 9.75	42.00 ± 16.36	0.156
4	54.17 ± 10.76	43.00 ± 21.04	0.181
6	56.33 ± 14.99	51.83 ± 6.55	0.590
8	54.67 ± 9.00	42.17 ± 18.37	0.134
10	48.50 ± 10.29	40.00 ± 13.56	0.308
12	53.83 ± 10.65	45.17 ± 15.35	0.299

After feeding time: 08:00 am (0), 10:00 am (2), etc. a,b,c,d Means within column bearing different superscripts differ significantly (p < 0.05),

The circulating progesterone and estrogen profiles were used as established indirect markers of ovarian activity in nutritional intervention studies, as these endocrine parameters have been shown to reflect underlying reproductive physiology even in the absence of direct fertility outcomes in buffalo cows fed roughages with differing estrogenic activity [25].

Table 9 presents the effects of MLP and sampling time on serum progesterone concentrations during the estrous cycle of female buffaloes. Results from the linear mixed-effects model showed that dietary treatments had no significant effect on progesterone concentration (β = −0.49 ± 0.48 ng/mL; p = 0.310), indicating that MLP did not alter luteal progesterone secretion. The estimated marginal means (EMMs) presented in Table 9 further confirmed the absence of dietary effects at all sampling points (p > 0.05). Progesterone concentrations remained consistently low (<1.5 ng/mL) in both groups, with comparable profiles from day 0 to day 21.

**Table 9:** Estimated marginal means of progesterone hormone level concentration by treatment and time.

Time (day)	Control (Mean ± SEM)	Treatment (Mean ± SEM)	Mean difference	p-value
0	1.41 ± 0.32	0.92 ± 0.36	−0.49	0.310
3	0.14 ± 0.36	0.37 ± 0.36	0.23	0.644
6	0.42 ± 0.42	0.38 ± 0.36	−0.04	0.399
9	0.86 ± 0.32	0.32 ± 0.42	−0.54	0.302
12	0.82 ± 0.29	0.99 ± 0.36	0.17	0.721
15	0.67 ± 0.42	0.81 ± 0.42	0.14	0.804
18	0.35 ± 0.51	0.39 ± 0.42	0.04	0.955
21	0.87 ± 0.42	1.22 ± 0.42	0.35	0.550

In contrast, sampling time had a significant effect on progesterone concentration, reflecting the expected physiological fluctuations across the estrous cycle. Progesterone levels declined significantly on day 3 (p < 0.05) and tended to decrease on day 6 (p = 0.059) compared with day 0, consistent with the early luteal regression phase following prostaglandin-induced estrus synchronization (Table 10). Importantly, no significant treatment × time interaction was detected (p > 0.05), indicating that the temporal pattern of progesterone secretion was similar between the control and legume-supplemented groups throughout the estrous cycle.

**Table 10:** Linear mixed-effects model results for progesterone hormone level concentration.

Fixed effects	Estimate (β)	Standard error	z-value	p-value	95% Confidence interval
Treatment (treatment vs control)	−0.49	0.48	−1.01	0.310	−1.44 to 0.46
Time (day)					
Day 3	−1.28	0.48	−2.64	0.008	−2.22 to −0.33
Day 6	−0.99	0.53	−1.89	0.059	−2.02 to 0.04
Day 9	−0.55	0.46	−1.21	0.227	−1.44 to 0.34
Day 12	−0.59	0.44	−1.35	0.178	−1.44 to 0.27
Day 15	−0.74	0.53	−1.42	0.156	−1.77 to 0.29
Day 18	−1.06	0.60	−1.77	0.077	−2.24 to 0.12
Day 21	−0.54	0.53	−1.02	0.306	−1.57 to 0.49
Treatment x Time				0.479	

Table 10 shows parallel progesterone trajectories between treatments, characterized by low concentrations during estrus and modest increases during the mid-luteal phase. Overall, the results indicate that increasing crude protein and amino acid intake through MLP supplementation did not modify progesterone dynamics in female buffaloes with adequate body condition and nutritional status.

Table 11 and Table 12 summarize the effects of dietary treatment and sampling time on serum estrogen concentrations during the estrous cycle of female buffaloes. Results from the linear mixed-effects model indicated that dietary treatment had no significant main effect on estrogen concentration (β = −0.25 ± 1.15 pg/mL; p > 0.05), indicating that supplementation with MLP in the concentrate did not directly alter circulating estrogen levels.

**Table 11:** Linear mixed-effects model results for estrogen hormone level concentration.

Fixed effects	Estimate (β)	Standard error	z-value	p-value	95% Confidence interval
Treatment (treatment vs control)	−0.25	1.15	−0.21	0.830	−2.51 to 2.01
Time (day)					
Day 3	5.17	1.15	4.48	<0.001	2.91 to 7.43
Day 6	1.86	1.15	1.61	0.107	−0.40 to 4.12
Day 9	4.13	1.02	4.05	<0.001	2.13 to 6.12
Day 12	0.54	1.02	0.53	0.594	−1.45 to 2.54
Day 15	2.84	0.94	3.01	0.003	0.99 to 4.68
Day 18	0.73	0.94	0.77	0.439	−1.12 to 2.58
Day 21	0.37	0.94	0.39	0.696	−1.48 to 2.22
Treatment x Time				<0.001	

**Table 12:** Estimated marginal means of estrogen hormone level concentration by treatment and time.

Time (day)	Control (Mean ± SEM)	Treatment (Mean ± SEM)	Mean difference	p-value
0	2.20 ± 0.67	1.95 ± 0.94	−0.25	0.830
3	7.37 ± 0.94	5.52 ± 0.67	−1.85	0.109
6	4.06 ± 0.94	4.33 ± 0.77	0.27	0.824
9	6.32 ± 0.77	5.08 ± 0.77	−1.24	0.253
12	2.74 ± 0.77	3.51 ± 0.94	0.77	0.528
15	5.03 ± 0.67	4.15 ± 0.67	−0.88	0.346
18	2.93 ± 0.67	1.29 ± 0.77	−1.64	0.108
21	2.56 ± 0.67	0.87 ± 0.77	−1.69	0.096

In contrast, sampling time exerted a highly significant effect on estrogen concentration (p < 0.001), reflecting marked temporal fluctuations throughout the estrous cycle (Table 11). Estrogen concentrations increased significantly on day 3 (p < 0.001) and day 9 (p < 0.001), with an additional significant rise observed on day 15 (p < 0.01) compared with day 0. These variations correspond to follicular development and regression phases following estrus synchronization.

The treatment × time interaction was not significant (p > 0.05 for all interaction terms), indicating that the temporal pattern of estrogen secretion did not differ between the control and legume-supplemented groups across the estrous cycle. Table 12 illustrates the estrogen hormone profiles, showing parallel cyclic patterns between dietary treatments, characterized by a pronounced peak around estrus (day 0), followed by secondary elevations during the mid-cycle. Overall, the results indicate that MLP did not significantly modify estrogen dynamics, and the observed fluctuations were primarily driven by physiological changes associated with the estrous cycle rather than dietary protein or amino acid intake. EMMs presented in Table 12 revealed that estrogen concentrations in the control group were numerically higher than those in the treatment group at most sampling points. However, none of these between-group differences were statistically significant (p > 0.05). The greatest numerical differences were observed on day 3 and day 21 between the control and treatment groups (p < 0.05).

## DISCUSSION

### Growth performance and body condition considerations

The diets provided in the study did not significantly improve the growth performance of buffalo. However, there was a 58.73% numerical improvement in weight gain compared to the buffalo control. Studies on buffaloes under explicit feed restriction and low body condition scores are very limited, but substantial evidence indicates that animals in nutritionally restricted systems or with poor body condition exhibit markedly different physiological and metabolic responses compared with well-fed, high-BCS animals. For example, in dairy cattle, short-term feed restriction was associated with increased feeding behaviors and metabolic profiles. Recent reviews in buffalo have demonstrated that feed restriction induces negative energy balance and profound alterations in metabolic and hormonal regulation [26]. The BCS has also been recognized as an important indicator of nutritional status and productive potential in buffaloes, with low BCS associated with impaired production and reproductive performance [27]. Hence, direct comparisons between the present study, conducted with buffaloes in good BCS with adequate nutrition, and studies involving feed-restricted or low BCS are inherently limited and should be interpreted with caution.

### Possible mechanisms for numerical improvement in weight gain

The improvement may be attributed to increased intake of crude protein, DM, and OM, as well as the proportion of the feed that was digested. The results showed that buffalo given legumes had better feed digestibility than the control. These important macronutrients can enhance the content of essential nutrients in buffalo, thereby increasing rumen metabolism [28]. The higher intake of crude protein and OM provided adequate substrate for rumen microbes to proliferate and easy digestibility, thereby improving the digestible component of the feed. Replacing concentrates with MLP increases feed protein content and digestibility. Protein derived from leguminous leaves is more easily degraded in the rumen than protein derived from grain feed ingredients originating from agricultural waste [29]. The average amount of feed offered to animals in the current study ranged from 2.3 to 2.35% of body weight. These amounts were slightly lower than the intake required by buffalo for maintenance, being 2.4-2.9% of body weight [27]. Nevertheless, both treatment and control animals consumed crude protein in amounts exceeding the maintenance requirements reported by Faraz and Waheed [20], with protein intake reaching up to 7.64 g per unit of metabolic body weight. Furthermore, digestible protein intake in the treatment group met the maintenance requirement, whereas animals in the control group exhibited values approximately 22% below the maintenance requirement suggested by Faraz and Waheed [20]. This difference may partly explain the divergent physiological responses observed between the experimental groups. Increasing the CP content of the diets due to the substitution of concentrate by MLP increased the intake of DM, OM, CP, and GE up to 6.9%, 7.8%, 26.87%, and 9.14% by female buffalo, respectively (*p* < 0.01). The results of the current study were in line with those of Abdel-Raheem *et al*. [28], who found that supplementing forage feed with various leguminous leaves increased intake and body weight gain. The CP intake was recorded at female buffalo receiving additional MLP for each kg of body weight (kg), being 19.3%, and metabolic body weight (kg BW0.75), being 24.6%, compared to those buffaloes receiving control diets. The results of the current study are similar to those reported by Akhtar *et al*. [30].

### Amino acid intake from MLP supplementation

The MLPs are rich in amino acids (Table 3), so supplementing the leaves with concentrates must increase the amino acid content of the treatment feed. In general, the supplementation of MLP to the diet increased the daily intake of most amino acids but not L-aspartic acid (Table 6). Most legume leaves are reported to include and have been used as sources of amino acids [29]. Female buffaloes had the four highest amino acid intakes in both groups: L-glutamic acid, L-aspartic acid, L-leucine, and L-arginine. In general, supplementation of MLP to the daily ration of female buffalo in treatment groups increased the animals’ daily amino acid intake. Since amino acids regulate key aspects of reproduction, such as gametogenesis, fertilization, implantation, placentation, and fetal growth and development, the presence of amino acids in diets is essential for animal reproduction [31]. Therefore, the inclusion of MLP in the diet is one strategy to improve the fertility and reproductive status of female buffalo.

### Effects of MLP supplementation on urea concentration and blood glucose

The postprandial (post-feeding) increase in serum urea can be used as an indicator of protein intake and absorption, as dietary protein is converted into urea by the liver and released into the bloodstream. The urea concentration in treatments and control shows no difference at all post-feeding times. The results of the current study were inconsistent with those of Naveed-ul-Haque *et al*. [32]. The current study demonstrated that MLP supplementation provided more protein and amino acids for degradation by rumen microbes, thereby increasing blood urea concentration. The decline in blood urea concentration observed at 6 h post-feeding may reflect the onset of urea utilization or increased excretion. Blood glucose is a source of energy, and its availability as circulating serum glucose could indicate increased energy availability from glucogenic VFA, or glucogenesis itself. Blood urea concentration was expressed as blood urea (mg/dl), not as blood urea nitrogen (BUN). The normal reference range for blood urea in adult buffaloes is approximately 12.84–57.78 mg/dl [33]. In this study, glucose levels peaked at 6 h and troughed at 10 h postprandially. In both cases, it indicates that their animals use glucose. This is because insulin availability during daytime glucose absorption by the muscle is influenced by insulin sensitivity [34]. The blood glucose concentration in the treatment group in the current study was not affected by increased CP intake. Our results are in line with the findings of Naveed-ul-Haque *et al*. [32], who reported that an increase in CP intake increases blood urea concentration but has no effect on blood glucose concentration. Moreover, protein tends to cause a gradual rise in blood glucose levels over several hours because it is metabolized more slowly than carbohydrates. Blood glucose concentration was expressed in mg/dL, with a normal reference range of 22.33–97.49 mg/dL for adult buffaloes under tropical conditions [33].

### Effects of MLP supplementation on reproductive hormone profile

By monitoring hormone profiles longitudinally within a single estrous cycle, the present study provides a more physiologically relevant assessment of dietary effects on ovarian function, an approach that remains infrequently used in controlled buffalo nutrition studies [35]. Therefore, this observation period was insufficient to reliably assess reproductive parameters, such as estrus duration, conception rate, and calving interval, which require extended longitudinal monitoring across multiple reproductive cycles, and was constrained by the availability of the animal facility. This study selected progesterone and estrogen as primary endocrine indicators because they directly reflect ovarian status and luteal activity in ruminants, whereas luteinizing hormone (LH), FSH, IGF-1, and insulin exhibit greater pulsatility or require intensive sampling to be interpreted meaningfully in nutritional studies [16, 25]. Progesterone and estrogen profiles have been used as reliable indicators of ovarian activity and luteal function in buffalo and cattle reproductive studies, with hormone concentrations corresponding to follicular dynamics and corpus luteum development as observed via ultrasonography [36, 37]. Overall, the level of progesterone hormone concentration in female buffalo during the estrous cycle did not differ between the treatment and the control groups (p > 0.05), which indicated that the addition of a protein diet in the treatment group had no significant effects on the estrous cycle in female buffalo. Although MLP supplementation in the treatment group increased the amino acid content of the diet, the level was still insufficient to exert a significant effect on reproductive hormones. These findings are inconsistent with those of Murikipudi *et al*. [15], who reported that dietary amino acids, both in absolute amounts and in balanced proportions, can influence reproductive function, particularly luteal function. This discrepancy may be explained by the relatively subtle role of amino acids in this context, as the nutritional requirements of both the treatment and control groups were already met, as indicated by an adequate BCS (≥3). The decrease and increase in progesterone levels in female buffalo are related to the development of the corpus luteum (CL) [38]. Our study showed fluctuations in progesterone concentration during one estrous cycle in both groups, indicating the development of CL. The highest progesterone concentrations occurred during 12-15 days and 9-12 days in the treatment and control groups, respectively. These findings are similar to those of a study by Sianturi *et al*. [24], which found that the progesterone level in buffaloes reaches its maximum between days 9 and 11, coinciding with the maximum size of the CL. In the current study, progesterone levels decreased earlier than those of Archunan [39]. Another study by Borghese *et al*. [2] found that plasma progesterone concentrations typically decrease 2 days before the onset of estrus (0.30 ng/mL) and remain at a minimal level on the day of estrus (0.09 ng/mL). In our study, the average concentration of progesterone is less than 1 ng/ml, as in studies with buffaloes by Archunan [39], while in Nili, Murrah, local, and crossbred buffaloes in Bangladesh, the average concentration of progesterone is above 1 ng/ml [40]. According to Hashem *et al*. [25], the progesterone concentration ranged from 0.09 to 2.04 ng/mL during the estrous cycle, which is higher than our results. The concentration of the progesterone hormone is also affected by nutrient intake. A review by Pehan *et al*. [8] reported that increasing daily protein intake increases progesterone concentration in female buffalo; however, our study found no significant effect of additional legumes on progesterone. Estrogen plays a crucial role in the female reproductive cycle, particularly in stimulating estrus behavior and triggering the release of LH. In animal production, estrus can be induced either naturally by periodically exposing females to a male until they come into heat or artificially using prostaglandins administered via intramuscular injection, which typically brings the animal into estrus within 72 h. In our study, estrogen concentrations peaked on two specific days during the estrous cycle: day 3 and day 9, indicating two waves of follicle development, consistent with Sianturi *et al*. [24]. MLP supplementation did not affect the estrous cycle. This result is consistent with the study of Chaudhary *et al*. [26], which showed that under many conditions (moderate intake, adapted animals, pasture supplementation), Leucaena does not disrupt estrous cycle length or sexual behavior in female cows. The present findings also agree with Atmojo *et al*. [41], who demonstrated that mixed feeding systems incorporating 30%–40% Calliandra in the diet exhibited optimal reproductive performance without adverse effects in ruminants. However, the previous studies [26, 41] differed from the findings of Wyse *et al*. [42], which reported that Leucaena affected ovarian cyclicity and prolonged the lifespan of the CL in cows. The differences among these studies might be due to the common presence of phytoestrogens in the legume leaves. As reported by Hashem *et al*. [25], phytoestrogens have an important influence on the estrous cycle in buffalo. However, proper processing methods, such as wilting or ensiling, can reduce phytoestrogen concentrations by 15-25%, making these legumes safer for reproductive livestock [43]. Unfortunately, direct analytical quantification of phytoestrogens was not conducted in the current study due to the absence of specific laboratory analysis capabilities, which often require advanced chromatographic technique. However, the legume leaves used in the current study were processed through sun-drying. This process might reduce the phytoestrogen content of the legume leaves [43]. Moreover, the present study used a mixed legume of *Leucaena*, *Calliandra*, and *Gliricidia* at 10%, which might preserve reproductive hormones. Common practice among farmers was a mixed feeding system incorporating 30%-40% fresh legume leaves into the daily ration [41]. Our study indicated that the use of legume leaves should be limited to about 10% in the sun-dried mixing without adverse effects in buffalo. Importantly, the feeding strategy incorporated locally available MLP, offering a sustainable, nutritionally rich, and cost-effective alternative to conventional commercial diets.

### Rationale for using a 1:1:1 mixture of legume leaves

The legume leaves of *G. sepium, L. leucocephala*, and *C. calothyrsus* were mixed in a 1:1:1 ratio because a legume-based feed supplement is justified by their complementary nutritional characteristics and effects on rumen metabolism. The *G. sepium* contributes balanced protein with good palatability, legume Leucaena leucocephala provides high crude protein and lysine content, and legume *Calliandra calothyrsus* supplies condensed tannins that partially protect dietary protein from ruminal degradation [29]. The mixture of three legume leaves in equal proportions allows the benefits of each species to be utilized without dominance of any single component. Moreover, this mixture promotes a balanced supply of rumen-degradable and rumen-undegradable protein, thereby supporting microbial protein synthesis and improving nitrogen utilization efficiency [44]. Another advantage of the balanced mixture among the three legumes is the dilution effect on anti-nutritional compounds, such as mimosine in *Leucaena leucocephala* and condensed tannins in *Calliandra calothyrsus*, thereby reducing potential negative effects on feed intake and nutrient digestibility [29]. In addition, using equal proportions provides a neutral, reproducible baseline ratio for experimental evaluation. From a practical perspective, the 1:1:1 ratio reflects realistic smallholder feeding systems, where the availability of *G. sepium, L. leucocephala*, and *C. calothyrsus* may vary seasonally [45]. Therefore, the 1:1:1 mixture offers both nutritional and methodological advantages for evaluating mixed-legume supplementation in ruminant diets.

### Insights from amino acid level analysis

Unlike most existing studies that interpret dietary protein effects based solely on crude protein intake, the present study provides deeper insight at the amino acid level, particularly regarding differential intake of glutamic acid, arginine, and leucine. While crude protein concentration is traditionally used as a convenient metric, it does not capture the true physiological roles of individual amino acids, since the animal’s requirement is ultimately for specific amino acids rather than aggregate nitrogen alone [32]. Glutamic acid and other non-essential amino acids are key substrates for rumen microbial metabolism and nitrogen recycling, whereas essential amino acids such as leucine, methionine, and tyrosine play important roles in protein synthesis, signaling pathways, and endocrine function, in addition to their contribution to total crude protein. Recent reviews emphasize that balancing rations for individual amino acids rather than total metabolizable protein can improve the interpretation of nutrient utilization and physiological outcomes in ruminants, including growth, metabolic status, and reproductive processes. By focusing on the pattern of specific amino acid intake rather than crude protein alone, this study advances understanding of protein nutrition in buffaloes and highlights the importance of amino acid composition for metabolic and endocrine responses in well-conditioned animals.

### Interpretation of hormonal responses and potential bioactive compounds

Protein and amino acid intake increased; however, these changes were not accompanied by consistent alterations in growth or progesterone levels, indicating that estrogen responses may be influenced by bioactive plant compounds present in MLP. Recent studies have reported that phytonutrients, including phytoestrogens and secondary plant metabolites, can interact with estrogen receptors and modulate estrogenic activity independently of nutritional energy balance [2, 17]. However, because phytonutrient concentrations were not directly quantified in the present study, this interpretation should be regarded as hypothesis-generating and warrants further investigation using targeted analytical and mechanistic approaches. Reproductive hormones such as progesterone and estrogen exhibit pronounced temporal variation across the estrous cycle, and single-time sampling may therefore fail to capture meaningful endocrine dynamics related to nutritional interventions [16]. By monitoring hormone profiles longitudinally within a single estrous cycle, the present study provides a more physiologically relevant assessment of dietary effects on ovarian function, an approach that remains infrequently used in controlled buffalo nutrition studies [16].

### Novelty and species-specific contributions

This study is among the few controlled investigations in swamp buffaloes that integrate dietary amino acid composition, postprandial metabolic responses, and estrous hormone dynamics. In contrast to the cattle- and dairy-buffalo–dominated literature, these findings provide species-specific physiological evidence relevant to swamp buffalo production systems [2, 16].

### Study limitations

This study has certain limitations that should be acknowledged. The relatively small sample size and short experimental duration may have limited the robustness of the findings. First, the relatively small sample size may have limited the statistical power to detect small effect sizes, particularly for highly variable endocrine parameters. However, the repeated measures experimental design and the use of mixed-effects models partially mitigated this limitation by increasing statistical efficiency and accounting for within-animal variation over time. Second, the experimental period was relatively short and focused primarily on metabolic and hormonal responses rather than long-term reproductive performance. As a result, reproductive outcomes such as estrus expression, conception rate, or pregnancy success were not evaluated. Hormone profiling was restricted to selected reproductive hormones, which may not fully capture the complexity of endocrine regulation underlying nutritional–reproductive interactions.

### Context-dependent nutritional effects and implications for future research

This study addresses a knowledge gap by focusing on well-conditioned swamp buffaloes (BCS > 3), whereas most previous nutrition–reproduction studies have primarily targeted undernourished or postpartum-anestrus cattle [16, 25]. By evaluating adequately conditioned animals, the findings demonstrate that dietary protein and amino acid enrichment do not necessarily enhance reproductive hormone responses, emphasizing the context-dependent nature of nutritional effects on buffalo reproduction. Moreover, the current study may exhibit metabolic and reproductive outcomes that differ from those of nutritionally stressed animals. While increased dietary protein is often associated with enhanced reproductive hormone responses under nutritional stress or feed restriction, such effects are context-dependent and influenced by baseline metabolic status in buffalo [27]. In well-conditioned buffaloes with adequate nutrition (BCS > 3), additional protein enrichment did not alter growth performance or circulating progesterone, suggesting that endocrine responses may plateau once nutrient requirements are met [27]. This observation challenges the common assumption that higher protein intake universally enhances reproductive hormones. Future studies should include larger animal populations and longer feeding periods to assess the long-term effects of legume leaf supplementation at supplementation levels exceeding 10% on reproductive performance, particularly estrus expression and conception, in animals with low body condition scores or under feed-limited conditions. Incorporating comprehensive reproductive assessments, including estrus monitoring, pregnancy diagnosis, extended hormonal profiling, and ultrasonographic evaluation of ovarian structures, would strengthen the interpretation of the effects of nutrition on reproductive physiology.

## CONCLUSION

Supplementation with mixed legume leaf powder (MLP; *G. sepium*, *C. calothyrsus*, and *L. leucocephala* in a 1:1:1 ratio) at 10.4% of dietary DM significantly increased nutrient intake and digestibility in female swamp buffaloes. Specifically, DM, OM, crude protein, and gross energy intakes rose by 6.9%, 7.8%, 26.87%, and 9.14%, respectively (p < 0.01), while crude protein digestibility and digested intakes of DM, OM, and crude protein were also improved (p < 0.05). Amino acid intake increased for 16 of the 17 measured amino acids (p < 0.05), except L-aspartic acid. Despite these improvements, MLP supplementation did not significantly affect body weight gain, serum urea or glucose concentrations (p > 0.05), or reproductive hormone profiles. Progesterone and estrogen concentrations throughout the estrous cycle did not differ significantly between the treatment and control groups (p > 0.05), and there was no treatment × time interaction.

These results indicate that under good nutritional management and adequate BCS (3.0–3.5), additional protein and amino acid supply from mixed legume leaf powder provides limited further benefits for growth performance or reproductive hormone dynamics in female swamp buffaloes. Farmers and nutritionists can therefore avoid unnecessary protein oversupply in well-managed herds, optimizing feed costs and reducing potential nitrogen excretion without compromising productivity. The 10% inclusion level of sun-dried mixed legume powder proved safe, with no adverse effects on estrous cyclicity or hormone profiles, supporting its use as a sustainable, locally available protein source in tropical smallholder systems.

The study employed a controlled CRD with detailed measurements of nutrient intake, apparent digestibility, amino acid profiles, postprandial blood metabolites, and longitudinal monitoring of reproductive hormones across a full estrous cycle. Use of linear mixed-effects models appropriately accounted for repeated measures, and blinded laboratory analysis minimized bias. The focus on adequately conditioned animals (good BCS and meeting maintenance requirements) addresses an important gap, as most prior research has targeted nutritionally stressed buffaloes.

The relatively small sample size (n = 16 after withdrawals) and short experimental duration (120 days, with hormone profiling limited to one estrous cycle) may have reduced statistical power to detect subtle effects on reproductive outcomes. Reproductive performance parameters such as estrus expression, conception rate, and calving interval were not evaluated due to facility constraints. Direct quantification of phytoestrogens was not performed, limiting the mechanistic interpretation of potential bioactive compounds.

Future studies should involve larger numbers of animals, longer feeding periods, and higher supplemen-tation levels (>10%) to assess long-term effects on reproductive performance. Investigations targeting buffaloes with low body condition scores, postpartum anestrus, or under feed restriction would better define the context-dependent benefits of mixed-legume supplementation. Comprehensive assessments that incorporate estrus behavior monitoring, pregnancy diagnosis, extended hormonal profiling, and ovarian ultrasonography would strengthen the understanding of the nutritional impacts on fertility. Additionally, quantification of phyto-estrogens and evaluation of different processing methods (wilting, ensiling) could clarify safety and efficacy across production systems.

In conclusion, while mixed legume leaf powder effectively enhances nutrient and amino acid supply and digestibility, it does not further improve growth or reproductive hormone profiles when female swamp buffaloes are already maintained under optimal nutritional conditions and good body condition. These findings emphasize the importance of precision feeding tailored to animals’ baseline status rather than blanket protein enrichment, contributing evidence-based guidance for sustainable buffalo production in tropical environments.

## DATA AVAILABILITY

The raw data supporting the findings of this study are available from the corresponding author upon reasonable request.

## AUTHORS’ CONTRIBUTIONS

YW: Conceptualization, investigation, methodology, formal analysis, and writing the original draft. LP: Methodology, investigation, formal analysis, data curation, and writing the original draft. DA: Conceptualization, methodology, investigation, collection of secondary data, and writing the original draft. TK: Conceptualization, validation, collection of secondary data, investigation, and writing the original draft. SA: Software, validation, investigation, data curation, and writing – review and editing. EH: Conceptualization, methodology, formal analysis, data curation, and writing the original draft. DY: Validation, formal analysis, collection of secondary data, data curation, and writing – review and editing. ER: Software, validation, formal analysis, collection of secondary data, and writing – review and editing. CT: Software, validation, collection of secondary data, data curation, and writing – review and editing. MC: Software, validation, formal analysis, collection of secondary data, and writing, review, and editing. PP: Conceptualization, methodology, formal analysis, data curation, and writing the original draft. AM: Validation, formal analysis, collection of secondary data, data curation, and writing – review and editing. All authors have read and approved the final manuscript.
